# Differential cellular responses associated with oxidative stress and cell fate decision under nitrate and phosphate limitations in *Thalassiosira pseudonana*: Comparative proteomics

**DOI:** 10.1371/journal.pone.0184849

**Published:** 2017-09-14

**Authors:** Qun Lin, Jun-Rong Liang, Qian-Qian Huang, Chun-Shan Luo, Donald M. Anderson, Chris Bowler, Chang-Ping Chen, Xue-Song Li, Ya-Hui Gao

**Affiliations:** 1 School of Pharmaceutical Sciences, Xiamen University, Xiamen, China; 2 School of Life Sciences, Xiamen University, Xiamen, China; 3 Key Laboratory of the Coastal and Wetland Ecosystems (Xiamen University), Ministry of Education, Xiamen, China; 4 Biology Department, Woods Hole Oceanographic Institution, Woods Hole, Massachusetts, United States of America; 5 Ecology and Evolutionary Biology Section, CNRS UMR8197 INSERM U1024, Institut de Biologie de l’Ecole Normale Supérieure (IBENS), Ecole Normale Supérieure, 46 rue d’Ulm, Paris, France; Stazione Zoologica Anton Dohrn, ITALY

## Abstract

Diatoms are important components of marine ecosystems and contribute greatly to the world’s primary production. Despite their important roles in ecosystems, the molecular basis of how diatoms cope with oxidative stress caused by nutrient fluctuations remains largely unknown. Here, an isobaric tags for relative and absolute quantitation (iTRAQ) proteomic method was coupled with a series of physiological and biochemical techniques to explore oxidative stress- and cell fate decision-related cellular and metabolic responses of the diatom *Thalassiosira pseudonana* to nitrate (N) and inorganic phosphate (P) stresses. A total of 1151 proteins were detected; 122 and 56 were significantly differentially expressed from control under N- and P-limited conditions, respectively. In N-limited cells, responsive proteins were related to reactive oxygen species (ROS) accumulation, oxidative stress responses and cell death, corresponding to a significant decrease in photosynthetic efficiency, marked intracellular ROS accumulation, and caspase-mediated programmed cell death activation. None of these responses were identified in P-limited cells; however, a significant up-regulation of alkaline phosphatase proteins was observed, which could be the major contributor for P-limited cells to cope with ambient P deficiency. These findings demonstrate that fundamentally different metabolic responses and cellular regulations are employed by the diatom in response to different nutrient stresses and to keep the cells viable.

## Introduction

Diatoms are a diverse group of unicellular eukaryotic microalgae that contribute greatly to the global climate, carbon cycling, and ocean ecosystem [1]. They thrive in a wide range of environments [2] and often form massive blooms as a result of their high productivity and efficient turnover rates [3]. Despite their success in marine ecosystems, the metabolic and cellular processes involved in their response to various habitat conditions, especially fluctuations in nutrient levels, remain largely unknown.

It has been suggested that diatoms possess numerous specific mechanisms for detecting and acclimating to nutrient stresses [2]. To date, many studies have focused on fundamental cellular responses of diatoms to nutrient stresses such as nitrogen (N) [4,5], phosphorus (P) [6,7], and silicon (Si) starvation and replenishment [8], and iron (Fe) stress [9–11]. For example, in the diatom *Thalassiosira pseudonana*, central carbon metabolism is adjusted in response to the onset of N starvation [4], and cellular acclimation responses to P deficiency include changes in cellular P allocation, increased P transport, and utilization of dissolved organic phosphorus [6]. However, there have been few molecular studies of how diatoms balance oxidative stress responses and cell death under nutrient stresses. Sophisticated, diatom species- and nutrient stress-specific cellular responses associated with reactive oxygen species (ROS) production and cell death decision were recently identified in response to Fe limitation in *T*. *pseudonana* [12,13] and to N limitation in *Phaeodactylum tricornutum* [3]. However, to our knowledge, very few studies have examined how a single diatom species responds to various types of nutrient stresses. Therefore, it is necessary to investigate the biochemical processes that contribute to cell death control, and the mechanisms underlying oxidative stress responses to different stresses, at the system level in diatoms.

One response of diatoms under nutrient stress is initiation of programmed cell death (PCD) [14–19]. The morphological, physiological and biochemical characteristics of PCD have been reported in several diatom species; for example, *Ditylum brightwellii* under N and P stress [20]; *Thalassiosira weissflogii* under N stress or exposure to exogenous aldehyde [21,22]; *T*. *pseudonan*a under Fe limitation [12,23]; *P*. *tricornutum* under N, or exogenous aldehyde stress [3,18,24]. However, the biochemical mechanisms underlying the activation and progress of PCD in diatoms remain elusive. Although there are many similarities in the characteristics of PCD across diatom species, some species- and nutrient-dependent features have been demonstrated. This variety may be attributed to different mechanisms, consistent with the suggestion that PCD comprises many diverse, genetically controlled, active cellular self-destruction pathways [17,25]. Therefore, it is necessary to investigate and compare the mechanisms of PCD triggered by different nutrient stresses and their roles in cell fate decision and stress acclimation in diatoms.

Previous studies in diatoms have suggested that, ROS response to nutrient stresses is usually related to oxidative stress and cell fate decision [3,13,18]. ROS are a series of highly reactive molecules considered to play key signaling roles in multiple cellular pathways, including adaptation and death [26]. A subset of putative genes and cellular metabolic pathways associated with ROS damage has been identified in cell survival and oxidative stress in Fe-limited *T*. *pseudonana* cells [12,13]. In *P*. *tricornutum*, a redox-sensitive signaling network has been proposed to mediate the cellular response to N stress, and subsequent cell survival [3]. Specifically, the redox potential of mitochondrial glutathione is strongly correlated with cell death triggered in response to the diatom-derived aldehyde decadienal [24]. However, knowledge of the molecular mechanisms associated with ROS production in response to adverse nutrient factors in diatoms remains poor. To understand oxidative stress responses and their involvement in cell death control in diatoms, it is necessary to investigate the cellular metabolic responses and adjustments associated with ROS production triggered by different nutrient stresses.

The use of isobaric tags for relative and absolute quantitation (iTRAQ) is well-suited to examine whole-cell proteomic changes at the time of harvest under different cultural conditions. Here, an iTRAQ-LC-MS/MS proteomics approach was used to evaluate relative protein abundances in *T*. *pseudonana* cultivated under nitrate or phosphate-limitation compared to nutrient-replete (control), respectively. The purpose of this study was to understand the different cellular metabolic mechanisms involved in the response to N and P stress, especially those associated with oxidative stress, cell viability and cell death. This integration of quantitative proteomics provides an in-depth view of cellular metabolic activity associated with ROS production and cell fate decision. The findings revealed two fundamentally different mechanisms associated with cell fate decisions that *T*. *pseudonana* cells employ under N- and P-limited conditions.

## Materials and methods

### Algal cultures

*T*. *pseudonana* CCMP1335 was obtained from the Provasoli-Guillard National Center for Marine Algae and Microbiota (NCMA; https://ncma.bigelow.org/). The cells were grown in axenic batch cultures with sterilized f/2-enriched medium and incubated at 18°C under a 12-h light/dark cycle at 60 μmol photons m^−2^ s^−1^. Middle exponentially growing cells (day 4, ~3.0 × 10^6^ cells mL^−1^) were harvested by centrifugation (3,000 × g, 20 min, 18°C), according to Bidle *et al*. [23], washed three times with autoclaved filtered seawater, and used as inoculum for triplicate 4-L treatments of (1) replete f/2 + Si seawater medium (control; 989.8 and 40.8 μM initial concentrations of N-NO_3_^−^ and P-PO_4_^3−^, respectively); (2) f/2 + Si seawater medium without added N-NO_3_^−^ (N-limited condition; 56.0 μM base N-NO_3_^−^ concentration); or (3) f/2 + Si seawater medium without added P-PO_4_^3−^ (P-limited condition; 0.59 μM base P-PO_4_^3−^ concentration). The inoculation density was 1.5 × 10^5^ cells mL^−1^. Autoclaved filtered seawater was prepared according to Li *et al*. [27]: A 0.45 μm pore size membrane was used to filter seawater, then filtered seawater was autoclaved.

### Analysis of physiological parameters

Cell abundance was determined at the same time daily under a microscope (Olympus BH-2, Japan) using a hemocytometer. The photochemical quantum yield of photosystem II (*F*_*v*_*/F*_*m*_) was measured in triplicate with an XE-PAM analyzer (Walz, Germany) in daily samples according to the manufacturer’s instructions.

Specific growth rate (μ, day^-1^) was calculated using the following equation [28]: μ = (ln N2 – ln N1)/(t2 – t1), where N_1_ and N_2_ were the cell densities at the time t_1_ and t_2_.

### Determination of nutrient concentrations

N-NO_3_^−^ and P-PO_4_^3−^ concentrations in the medium were measured daily in triplicate using classic colorimetric methods (Technicon AutoAnalyzer AA3; Bran+Luebbe GmbH, Germany) according to the manufacturer’s instructions. N-NO_3_^−^ was analyzed using the copper-cadmium column reduction method (Method No. G-172-96 Rev.7; Bran+Luebbe GmbH, Germany). P-PO_4_^3−^ was determined via the molybdenum blue method (Method No. G-175-96 Rev.8; Bran+Luebbe GmbH, Germany). The detection limits for N-NO_3_^−^ and P-PO_4_^3−^ were 0.015 and 0.024 μM respectively. Intracellular particulate phosphorus (PP) was measured daily in triplicate. Cells were collected on pre-combusted GF/F filters membrane (450°C, 2h), digested with magnesium sulfate (MgSO_4_) for 2h, and determined via the method described previously [29].

### Determination of chlorophyll a concentration

Photosynthetic pigments were extracted using 90% acetone and kept at 4°C for 24 h in darkness. The A664 and A630 values of the extract were determined in triplicate using a spectrophotometer. Chlorophyll a concentration was calculated using the following equation [30]: chlorophyll a = 11.47 × A664 – 0.40 × A630.

### Identification of neutral lipid accumulation

Qualitative neutral lipid determination with boron-dipyrromethene (Bodipy 505/515) was performed in triplicate as described previously [31]. Briefly, cells were collected by filtration through a membrane (2 μm pore size) and washed twice with PBS (0.1 M) to remove the residual medium. The pellets were fixed with 4% formaldehyde solution (prepared with autoclaved filtered seawater, pH 7.4) for 30 min and subsequently washed three times with 0.1 M PBS. The cells were then stained with Bodipy 505/515 working solution for 15 min at room temperature, washed with 0.1 M PBS and observed via fluorescence microscopy (Olympus, Japan).

### In vivo cell staining and flow cytometry

Cells were collected via centrifugation (10,000 × g, 10 min, 4°C), divided into four sub-samples, and treated as followed respectively: (1) re-suspended in autoclaved filtered seawater, and incubated with 5-(and-6)-chloromethyl-2′,7′-dichlorodihydrofluorescein diacetate, acetyl ester (CM-H_2_DCFDA, 5 mM; Invitrogen, Thermo Fisher Scientific, Waltham, MA, USA) to determine the cellular ROS level; (2) re-suspended in phosphate-buffered saline (PBS, pH 7.4), and incubated with Z-VAD-FMK-FITC in situ marker (CaspACE; 20 μM; Promega, Madison, WI, USA) to determine activated caspase levels; (3) re-suspended in Annexin binding buffer (10 mM HEPES, 140 mM NaCl, and 2.5 mM CaCl_2_, pH 7.4), and incubated with Annexin V (10 μL per 100 μL cells; Invitrogen) to determine the externalization of phosphatidylserine; and (4) re-suspended in autoclaved filtered seawater, and incubated with SYTOX Green nucleic acid stain (1 μM; Invitrogen) to visualize dead cells. All samples were incubated for 20 min at room temperature in the dark. After harvested by centrifugation (10,000 × g, 10 min, 4°C), cells were washed three times with specific buffer (autoclaved filtered seawater for CM-H_2_DCFDA and SYTOX Green nucleic acid stain; 1×PBS for CaspACE and Annexin V), and re-suspended in 1 mL of autoclaved filtered seawater (CM-H_2_DCFDA and SYTOX Green nucleic acid stain) or 1×PBS/2% formalin (CaspACE and Annexin V).

The portion of positively stained cells per 10^4^ counted was determined at 520 nm after excitation with a 488 nm laser through an InFlux Model 209S Mariner flow cytometer (BD Fortessa, USA). Channels for relative staining chemicals were selected on the software. Gating and data analysis were performed using FlowJo software. Student’s t and Duncan’s multiple range tests were applied to assess group differences in discrete variables using SPSS version 18.0 (SPSS Inc, Chicago, IL, USA). P < 0.05 was considered statistically significant.

### RNA extraction and quantitative PCR

Total RNA was isolated by standard phenol/chloroform extraction. Briefly, approximately 10^7^ cells were filtered through a membrane (2 μm pore size), scrunched into powder in liquid nitrogen, immediately transferred into 1 mL of Trizol phenol (Invitrogen, Life Technologies, Waltham, MA, USA) and vortexed for 3–5 min at room temperature prior to centrifugation (12,000 × g, 10 min, 4°C). The supernatant was transferred into a new tube. Chloroform (200 μL) was added to the sample. The mixture was vortexed for more than 20 s, allowed to stand at room temperature for 15 min and centrifuged (12,000 × g, 10 min, 4°C). The upper layer was carefully transferred into another tube, mixed with an equal volume of isopropanol (~500 μL), and allowed to react at room temp for 10 min. The RNA product was obtained by centrifugation (12,000 × g, 10 min, 4°C), washed twice with 75% ethanol prepared with DEPC-H_2_O and dried for several minutes at room temperature. The pellet was dissolved in a proper volume of DEPC-H_2_O (30–100 μL) and synthesized into cDNA for gene expression level quantification.

One μg of total RNA, pre-treated by RQ1 RNase-Free DNase (Promega, Madison, WI, USA), was reverse-transcribed into the first strand of cDNA using random hexamers (Promega) and Improm-II reverse transcriptase (Promega) at 25°C for 10 min and 48°C for 60 min. Expressions of the selected genes were determined via Quantitative PCR analysis performed for three independent biological samples using a Rotor-Gene 6000 system (Corbett, Mortlake, Australia). The gene-specific primers were designed using Primer-BLAST [32]. Quantitative PCRs were initiated via the addition of cDNA fragments to 1×SYBR green PCR master mix (Promega), which contained 30 nM of forward and reverse gene-specific primers. The PCR cycles for the conditions were set to 95°C for 20 s for 1 cycle, 95°C for 10 s, 60°C for 20 s and 72°C for 20 s for 45 cycles. The fluorescence intensity from the SYBR green/double-stranded PCR product complex was continuously monitored from cycles 1 to 40. Each gene was detected in triplicate with normalization to the geometric mean of the two internal controls (translation elongation factor-1α [Thaps3_29435] and ubiquitin-conjugating enzyme [Thaps3_27711]), and calibrated to its expression level in the control cells using the Bio-Rad CFX Manager 3.1 software. Reactions with no template or DNase-treated RNA were run for each gene as controls.

To select the best internal controls, six housekeeping genes, including translation elongation factor-1α, ubiquitin-conjugating enzyme, actin, gluceraldehyde-3-phosphate dehydrogenase, tubulin, and ubiquitin were selected as candidate reference genes. Their Ct values were obtained by quantitative PCR, and their stabilities were analyzed by Delta Ct, geNorm (ver. 3.5), and Normfinder (ver. 0.953) to find the best reference genes. Details of primers sequences, primer efficiencies, correlation factors, and results of geNorm and Normfinder were showed in S1 Table.

### Protein extraction and quantification

Proteins were extracted as described previously [8]. In brief, 1 L of control, N-limited or P-limited sample was filtered through a membrane (2 μm pore size). The collected cells were resuspended with 10 mL of medium into a 15 mL tube. Following centrifugation (3,000 × g, 10 min, 4°C), the supernatant was discarded and the cell pellet resuspended in 10 mL of TRIzol Reagent (Invitrogen, Life Technologies, Waltham, MA, USA). The proteins were subsequently extracted according to the manufacturer’s recommendations. At the final step, the protein pellet was resuspended in an appropriate volume of lysis buffer (8 M urea, 0.1 M Tris/HCl, pH 8.0) and incubated at room temperature for 60 min. After centrifugation (12,000 × g, 30 min, 4°C), the supernatant was collected.

An aliquot of the supernatant was taken to determine the total protein concentration using a Bradford protein assay kit from Bio-Rad (Hercules, CA, USA) according to the manufacturer’s instructions. The remaining supernatant was kept at −80°C for further analysis.

### iTRAQ labeling

For each sample, a reduction step with DL-Dithiothreitol, and alkylation with iodoacetamide were followed by digesting 100 μg of protein at 37°C for 16 h using Trypsin Gold (Promega, Madison, WI, USA) with a protein: trypsin mass ratio of 30:1. The digested peptides were then dried by vacuum centrifugation and reconstituted in 0.5 M triethylammonium bicarbonate (Applied Biosystems, Milan, Italy).

Six samples (two of each condition: control, N-limited and P-limited) were labeled with different iTRAQ tags using an iTRAQ Reagent 8-plex Kit (Applied Biosystems, Foster City, CA, USA) according to the manufacturer’s protocol. The samples were labeled as follows: iTRAQ tags 113 and 114 for control samples; tags 115 and 116 for N-limited samples; and tags 117 and 118 for P-limited samples. The labeled peptides were incubated at room temperature for 2 h and then dried via vacuum centrifugation.

### LC-ESI-MS/MS analysis

Strong cationic exchange (SCX) chromatography was performed with a Shimadzu LC-20AB HPLC pump system (Shimadzu, Kyoto, Japan). The labeled replicates were pooled 1:1, reconstituted in 4 mL of buffer A (25 mM NaH_2_PO_4_ in 25% Acetonitrile, pH 2.7) and then loaded onto a 4.6 × 250 mm Ultremex SCX column containing 5-μm particles (Phenomenex, Torrance, CA, USA). The fractionated peptides were eluted at a flow rate of 1 mL/min with the following buffer gradient: 100% buffer A for 10 min; 5%–60% buffer B (25 mM NaH_2_PO_4_, 1 M KCl in 25% Acetonitrile, pH 2.7) for 27 min, and 60%–100% buffer B for 1 min. The system was maintained at 100% buffer B for 1 min, followed by equilibration with 100% buffer A for 10 min. The chromatograms were recorded at 214 nm, and fractions were collected every minute. The eluted peptides were subsequently pooled into 20 fractions, desalted with a Strata X C18 column (Phenomenex), vacuum-dried, and reconstituted in 0.1% formic acid for LC-MS/MS analysis.

For LC-ESI-MS/MS analysis using a Q Exactive (Thermo Fisher Scientific, San Jose, CA, USA), each fraction was resuspended in solution A (2% Acetonitrile, 0.1% formic acid) and centrifuged at 20,000 × g for 10 min to obtain peptides with an average final concentration of approximately 0.5 μg/μL. A 10 μL volume of supernatant was collected to load onto an LC-20AD nano HPLC (Shimadzu, Kyoto, Japan) through an autosampler to a C18 trap column. The peptides were then separated using a nanobored C18 column with a PicoFrit nanospray tip (inner diameter, 75 μm; New Objectives, Woburn, MA, USA). The sample collection was performed at a rate of 8 μL/min for 4 min. The following gradient was run at 300 nL/min: 2%–35% solution B (98% Acetonitrile, 0.1% formic acid) for 44 min, linear gradient to 80% solution B for 2 min, maintenance at 80% solution B for 4 min, and a return to 5% solution B in 1 min.

The peptide samples were then subjected to nanoelectrospray ionization for tandem mass spectrometry (MS/MS) in a Q Exactive, which was coupled online to the HPLC system. Intact peptides were detected in the orbitrap at a resolution of 70,000 (more details can be referred to on the website of http://www.biotech.wisc.edu/services/massspec/instrumentationoverview/orbitrap). Peptides were selected for MS/MS using the high-energy collision dissociation operating mode with a normalized collision energy (NCE) setting of 27.0 and a stepped NCE of 12.0%. The ion fragments were detected in the orbitrap at a resolution of 17,500. A data-dependent procedure that alternated between one MS scan and 15 MS/MS scans was applied for the 15 most abundant precursor ions above a threshold ion count of 20,000 in the MS survey scan, with a subsequent dynamic exclusion duration of 15 s. The applied electrospray voltage was 1.6 kV. Automatic gain control (AGC) was used to optimize the spectra generated by the orbitrap. The AGC target was 3e6 for full MS and 1e5 for MS2. For the MS scans, the m/z scan range was 350–2000 Da. For the MS2 scans, the m/z scan range was 100–1800.

### Proteomic data analysis

Raw data files acquired from the orbitrap were converted into MGF files using Proteome Discoverer 1.2 (Thermo Fisher Scientific, San Jose, CA, USA). Peptide and protein identification was performed via the Mascot search engine (version 2.3.02; Matrix Science, London, UK) against the database of the *T*. *pseudonana* genome. The genome was downloaded from NCBI and the Joint Genome Institute database (http://genome.jgi-psf.org/Thaps3/Thaps3.home.html, downloaded Oct. 2012; 34,736 sequences, including the Thaps3 finished chromosome data and Thaps3_bd unmapped sequence data [386 sequences]).

For protein identification, a mass tolerance of 0.05 Da was permitted for intact peptide masses and ±0.1 Da for fragmented ions, with an allowance for one missed cleavage in the trypsin digests. Gln->pyro-Glu (N-term Q), Oxidation (M), and Deamidated (NQ) were the potential variable modifications, and Carbamidomethyl (C), iTRAQ 8plex (N-term), and iTRAQ 8plex (K) were the fixed modifications. Peptide charge states were set to +2 and +3. To reduce the probability of false peptide identification, only peptides at the 95% confidence interval as determined by Mascot probability analysis greater than “identity” were counted as identified.

For relative protein quantification, proteins were selected for further analysis based on the following two criteria: 1) one protein had at least two confident unique peptides; 2) the cut-off value between replicates was smaller than 0.30. The quantitative protein ratios were weighted and normalized by the median ratio in Mascot according to the procedures in http://www.matrixscience.com/help/quant_statistics_help.html. Only unique peptides were used to quantify proteins. Student’s *t*-tests were performed using Mascot 2.3.02 software and *p* values < 0.05 were reported with an asterisk. To be identified as differentially expressed, a protein had to be quantified with at least three spectra to allow generation of a *p* value. Proteins with a ≥ 1.5-fold change between the N-limited or P-limited and control samples, and *p* values < 0.05, were determined as significantly differentially expressed.

### Function annotation

Clusters of Orthologous Groups (COGs, http://www.geneontology.org) were used to predict protein function, functional classification and statistics according to a previously reported method [33,34]. For the differentially regulated proteins, Gene Ontology (GO) enrichment analysis was performed to identify the affected cellular metabolic process. Functional annotations were processed using the Blast2GO program against the non-redundant protein database (NR; NCBI). The KEGG pathway database (http://www.genome.jp/kegg/) was used for protein function and interaction analyses in identical metabolic pathways. The COG description of each protein was prioritized. For proteins without a COG description, the NCBI and Uniprot_Swissprot descriptions, with consideration of KEGG pathway analysis results, were referred to with specific comments.

## Results

### General physiological and biochemical responses

**Fig 1:**
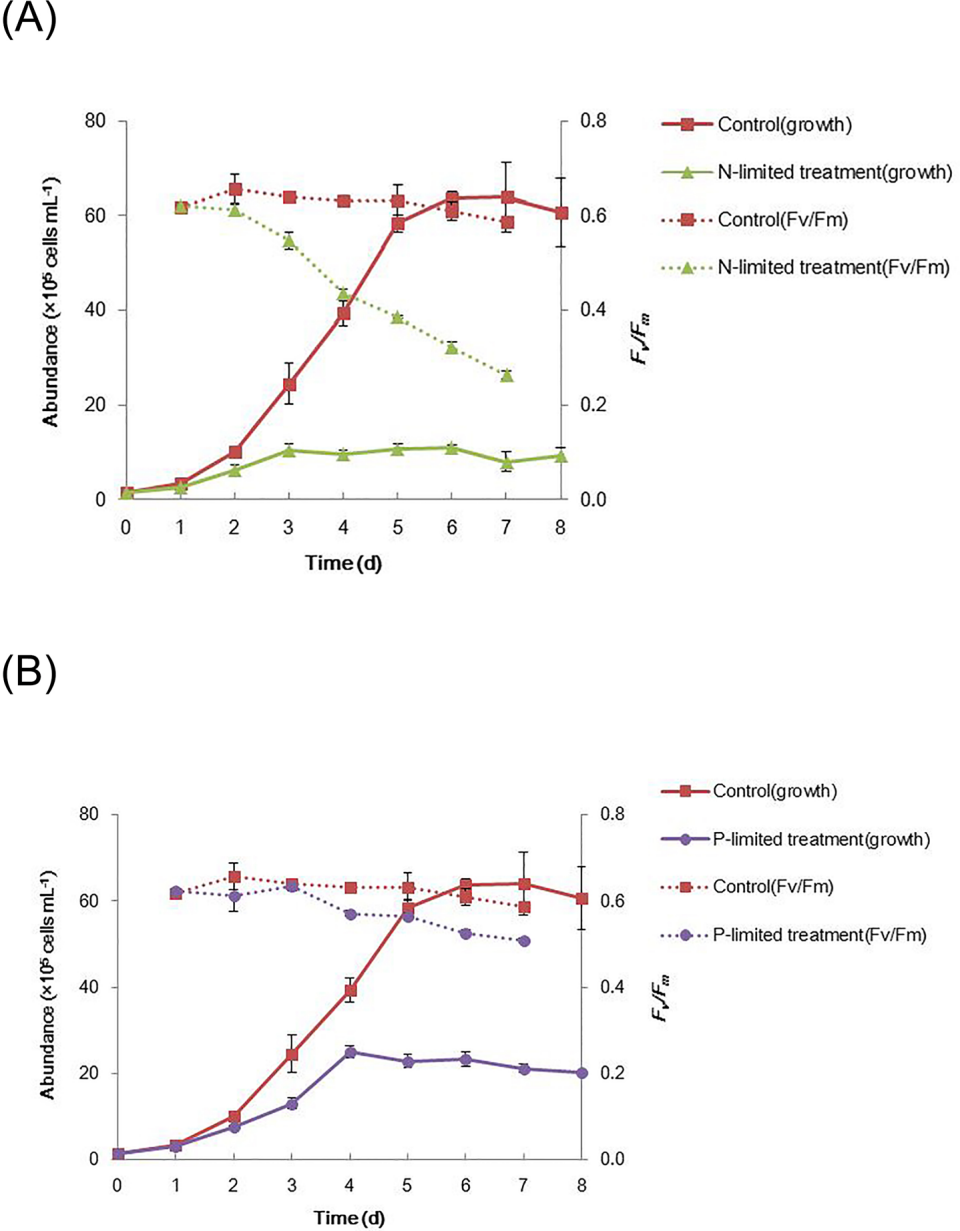
Cell growth (abundance, left vertical axis) and photosynthetic efficiency (*F*_*v*_*/F*_*m*_, right vertical axis) of N-limited (A) and P-limited (B) *T*. *pseudonana* cultures (mean ± SD of triplicate measurements).

**Fig 2:**
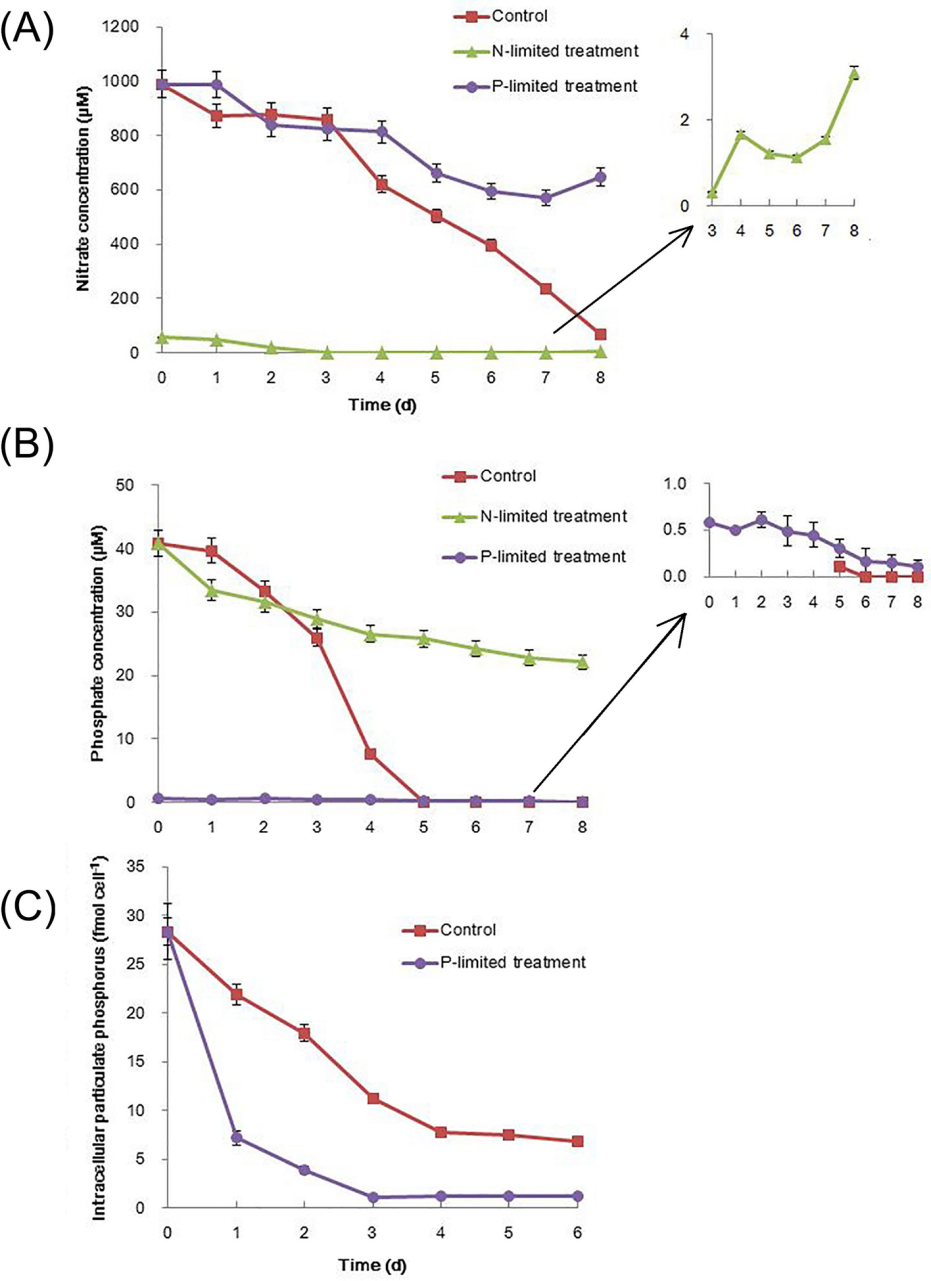
Concentrations of N-NO_3_^−^ (A) and P-PO_4_^3−^ (B) in media when *T*. *pseudonana* cells were cultured under control, N-limited, and P-limited conditions from days 0 to 8. Concentrations of intracellular phosphorus indicated as particulate phosphorus (C) in control and P-limited *T*. *pseudonana* cells from days 0 to 6. The error bars represent the standard errors from triplicate measurements (mean±SD).

When *T*. *pseudonana* cells were grown under nitrate-limited (N-limited) condition, there was a significant inhibition of maximum cell abundance, specific growth rate (μ, day^−1^), and photochemical quantum yield of photosystem II (PS II; *F*_*v*_*/F*_*m*_; Fig 1A). The N-limited *T*. *pseudonana* cultures reached stationary phase on day 3, corresponding to near-depletion of nitrate (NO_3_^-^) in the medium from day 3 (Fig 2A). The maximum cell abundance in N-limited cultures (~1.1 × 10^6^ cells mL^−1^) was 83% lower compared to control (~6.4 × 10^6^ cells mL^−1^). Compared to control μ (0.39 day^−1^), N-limited cultures had a 70% lower μ (0.12 day^−1^) on day 4 (Table 1). A substantial decrease in *F*_*v*_*/F*_*m*_ in N-limited cultures started at day 3 and a 30% lower *F*_*v*_*/F*_*m*_ (~0.44 versus ~0.63 in control) was observed on day 4 (Fig 1A; Table 1). N-limited cultures also showed great reductions by day 4 in cell volume, chlorophyll per cell and per cell volume, and protein content per cell and per cell volume (Table 1).

**Table 1:** General physiological and biochemical characteristics of N-limited, P-limited, and control *T*. *pseudonana* cells on day 4.

Parameter	Control	N-limited	P-limited
Growth rate	0.39±0.04	0.12±0.001	-0.09±0.005
Cell abundance, ×10^5^ per mL	39.3±2.75	9.5±1.02	25.0±1.32
*F*_*v*_*/F*_*m*_	0.63±0.01	0.44±0.006	0.57±0.005
Cell diameter, μm	5.20±0.32	4.63±0.48	5.13±0.31
Cell volume, μm^3^	93.75±17.10	68.97±17.38	87.28±16.38
Chl a per cell, pg per cell	0.39	0.22	0.32
Chl a per volume, fg per μm^3^	4.2	3.2	3.7
Protein content per cell, pg per cell	56.20	34.32	42.52
Protein content per volume, pg per μm^3^	0.60	0.50	0.49

Expectedly, P-limited also induced an obvious inhibition of maximum cell abundance, when they exposed to very low concentration of phosphate (PO_4_^3-^, 0.59 μM) at the beginning of cultural time (Figs 1B and 2B). The P-limited cultures reached stationary phase on day 4, with a 61% lower maximum cell abundance (~2.5 × 10^6^ cells mL^−1^) compared to control (day 6, ~6.4 × 10^6^ cells mL^−1^, Fig 1B). Different from N-limited culture, even though a gentler decrease in *F*_*v*_*/F*_*m*_ was detected in P-limited cultures on day 4 (only 10% lower *F*_*v*_*/F*_*m*_, ~0.57 versus ~0.63 in control), the value of *F*_*v*_*/F*_*m*_ was still higher than 0.5 (Fig 1B; Table 1), indicative of healthy photosystem II photochemistry [23]. However, the consumption of PO_4_^3-^ in the medium was only 0.14 μM from day 0–4 (Fig 2B), which obviously lower than the amount of PO_4_^3-^ utilization in N-limited and control cultures (14.23 and 33.27 μM, respectively; Fig 2B). Actually, during the whole cultural time (8 days), only 0.48 μM PO_4_^3-^ was consumed in P-limited cultures. Therefore, it is fair to say that phosphate was almost not being used for the growth in this P-limited culture. Consumption of intracellular phosphorus indicated as particulate phosphorus (PP) showed that intracellular phosphorus could be one way employed to support the phosphorus need for the growth. And intracellular PP was almost depleted by day 3 (Fig 2C), which may contribute to the status of the stationary phase in P-limited cultures by day 4.

**Fig 3:**
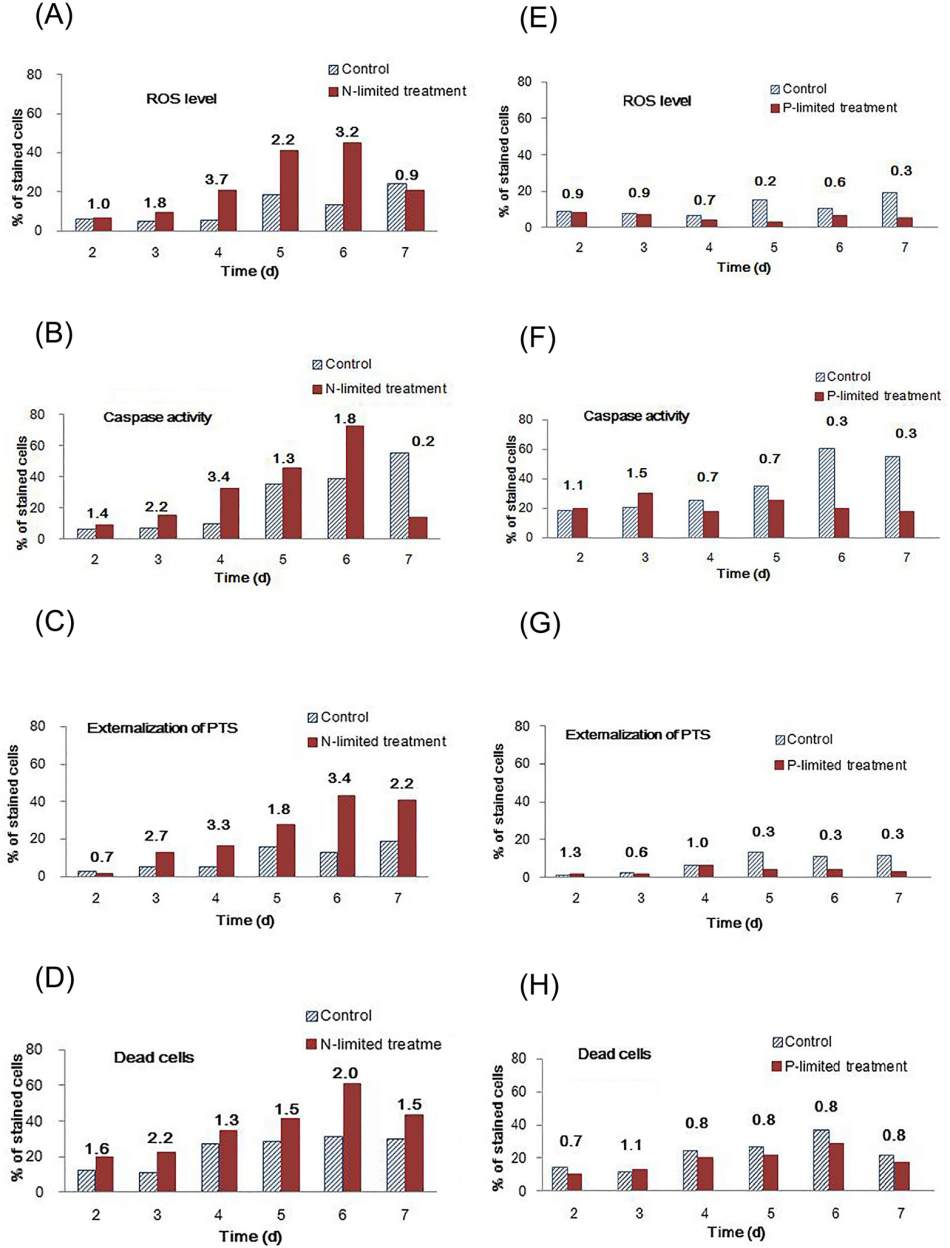
Examination of ROS production, biochemical characteristics of programmed cell death (PCD), and cell death in *T*. *pseudonana* cells grown under different conditions. *In vivo* detection of the ROS level (CM-H_2_DCFDA; A, E), caspase activity (CaspACE; B, F), externalization of phosphatidylserine (Annexin V; C, G), and dead cells (SYTOX; D, H) in *T*. *pseudonana* using flow cytometry. Cells were grown in nutrient replete (control), N-limited (A-D), or P-limited (E-H) media on days 2–7. Numbers above columns represent ratios of positive cells under limited: control conditions.

Diagnostic staining with CM-H_2_DCFDA confirmed N limitation induced *in vivo* oxidative stress in *T*. *pseudonana* cells (Fig 3A), with the greatest difference (3.7-fold) observed on day 4 in the N-limited condition (20.8%) compared to the control condition (5.7%; Fig 3A), although the percentage of cells positively stained for intracellular ROS was not the highest on this day. Considering aging is another main reason to induce ROS accumulation and PCD activation in *T*. *pseudonana* [23], the obviously increased percentages of cells positively stained for ROS on days 5 and 6 will not be taken into account in the present study. Therefore, it indicates that N limitation caused the greatest increase in ROS on day 4. Correspondingly, N-limited cultures showed a higher percentage of cells positive for typical PCD markers, caspase activity [35] (32.7%, 3.4-fold) and externalization of phosphatidylserine [36] (16.7%, 3.3-fold), both with the greatest difference in N-limited cultures compared to control cultures (Fig 3B and 3C) on day 4; this indicates a strong relationship between oxidative stress and PCD induced by N limitation. The ratio of dead cells was not significantly different between N-limited and control cultures on day 4 (Fig 3D), suggesting that some cells undergoing PCD due to oxidative stress were not yet dead.

Differently, no obvious *in vivo* ROS production or PCD activation were triggered in P-limited cells (mostly < 1.0-fold change versus control; Fig 3E–3G) for the duration of culture according to ROS and PCD diagnostic maker evaluation. This finding indicates that unlike N limitation, P-limited cultures did not experience oxidative stress or PCD within the time course of the experiment, even though they were exposed to low P-stress at the beginning of cultural time. Although no obvious ROS production and PCD activation were observed in P-limited cultures, the level of ROS still varied within the time course of the experiment. Relatively, the strongest ROS response and most obvious PCD characteristic in P-limited cultures (compared to control cultures) were assessed both on day 4 (0.7-fold of ROS and caspase activity, 1.0-fold of externalization of PTS), if we take no account of relative higher ROS production or PCD activation detected at the first days (days 2 and 3), which mainly due to that the cells just be transferred to the new environment.

Overall, the primary goal of this study was to investigate cellular responses associated with oxidative stress and cell fate decision under nitrate and phosphate limitations. The most acute oxidative stress and PCD activation in N-limited and relative higher level of ROS in P-limited cells both occurred on day 4; this time point was therefore selected for quantitative proteomic analysis sampling. Although there is likely to be a range of differences between the two growth stages, it was nevertheless expected that the major physiological differences between cultures would be due to changes in oxidative stress and cell death response.

### General results from the iTRAQ-based proteomic analysis

**Fig 4:**
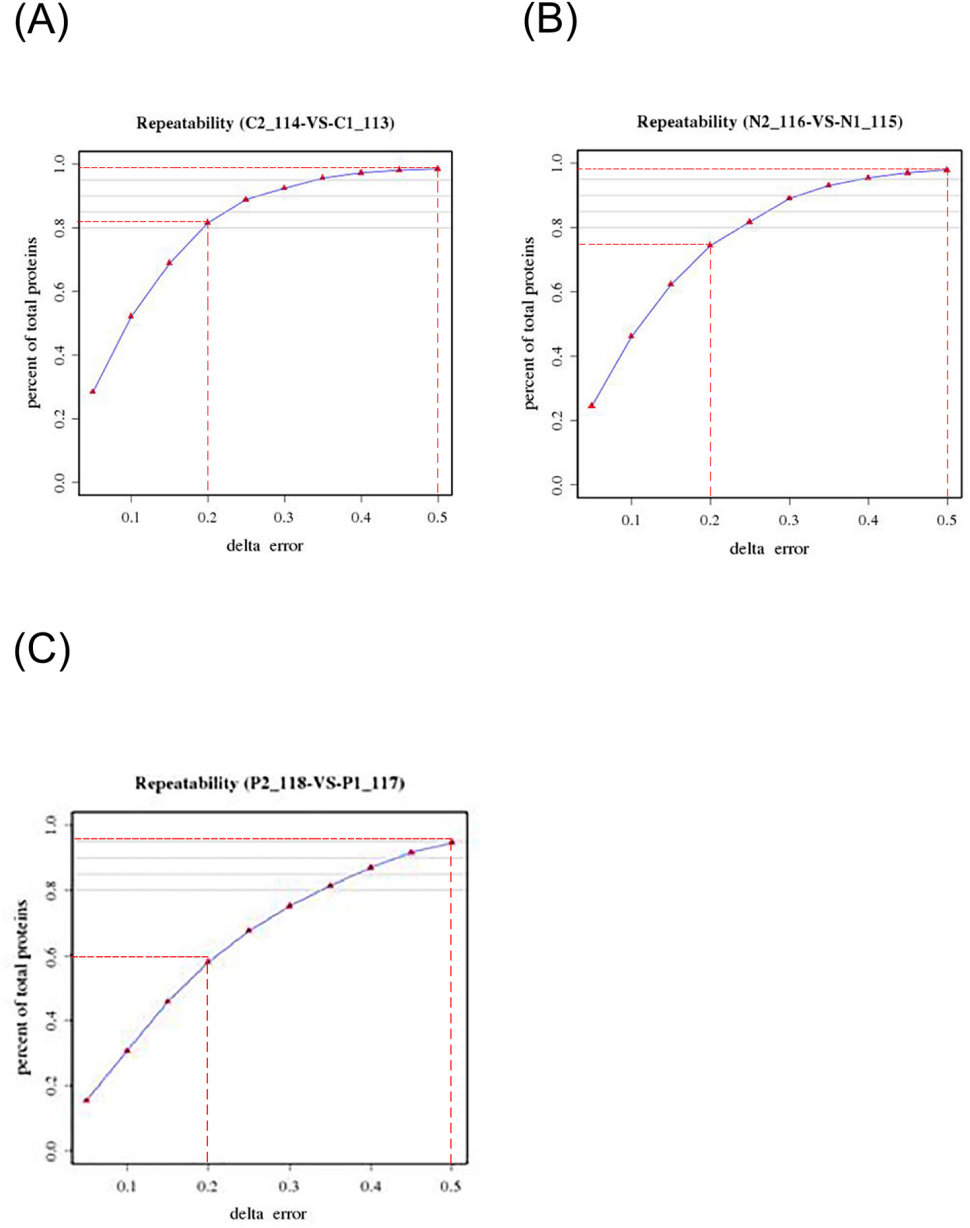
Good reproducibility between two biological replicates for each cultural condition, which indicated by the data that 95% of the proteins had differences with a delta error of less than 0.5 between two samples in all three conditions. (A) Control conditions; (B) N-limited culture conditions; (C) P-limited culture conditions.

To unravel the cellular metabolic responses associated with different levels of ROS production and cell death responses in N- and P-limited conditions, a well-developed iTRAQ-based proteomic approach was performed. Two biological replicates from N-limited, P-limited and control samples on day 4 in culture were used for proteomic analysis. The reproducibility of the analysis was evaluated by comparing the differences between two biological replicates of each condition using the Pearson correlation coefficient. More than 75% of the proteins had differences with a delta error of less than 0.2, and more than 95% had differences of less than 0.5, between control samples 1 and 2 or N-limited samples 1 and 2. Between P-limited samples 1 and 2, approximately 60% of the proteins had differences of less than 0.2, and 95% had differences of less than 0.5 (Fig 4). This indicates satisfactory reproducibility of the proteomic analysis.

In total, 1,151 unique putative proteins were identified in our samples, representing approximately 10% of the 11,390 predicted proteins in the *T*. *pseudonana* genome (S2 Table). Of these, 885 proteins were identified as changed in abundance in N-limited *T*. *pseudonana* compared to control (Table 2; S3 Table); whereas 886 changed proteins were identified in P-limited cells compared to control (Table 2; S4 Table). Significant changes in protein abundance were defined using a cutoff of 1.5-fold change and a *p*-value < 0.05 (based on a *t*-test). There were 122 significantly differentially expressed proteins (SDEPs) in N-limited cells (16 increased, 106 decreased) and 56 (9 increased, 47 decreased) in P-limited cells (Table 2; S5 and S6 Tables). Cells under both nutrient-limited conditions shared 28 SDEPs with reduced abundance; no proteins with increased abundance were shared. Among the shared proteins, 9 are involved in DNA replication, transcription, and translation, and protein maturation and secretion processes; their decreased expression corresponds to the reduced growth rates of *T*. *pseudonana* cultures under both nutrient-limited conditions.

**Table 2:** Summary of numbers of identified proteins under different conditions on day 4.

	Parameter	N-limitation	P-limitation
Proteins detected with changed abundance	Total number	855	866
	Number of hypothetical and predicted protein	235	235
	Number of protein with known function (% of total number)	647(73.1%)	648 (73.1%)
Significantly changed proteins with increased abundance	Total number	16	9
	Number of hypothetical and predicted protein	5	1
	Number of protein with known function (% of total number)	11(68.6%)	8(88.9%)
Significantly changed proteins with decreased abundance	Total number	106	47
	Number of hypothetical and predicted protein	31	22
	Number of protein with known function (% of total number)	75(70.8%)	25(53.2%)
Significantly changed proteins detected in both conditions	Total number	28	
	Number of hypothetical and predicted protein	11	
	Number of protein with known function (% of total number)	17(60.7%)	

**Fig 5:**
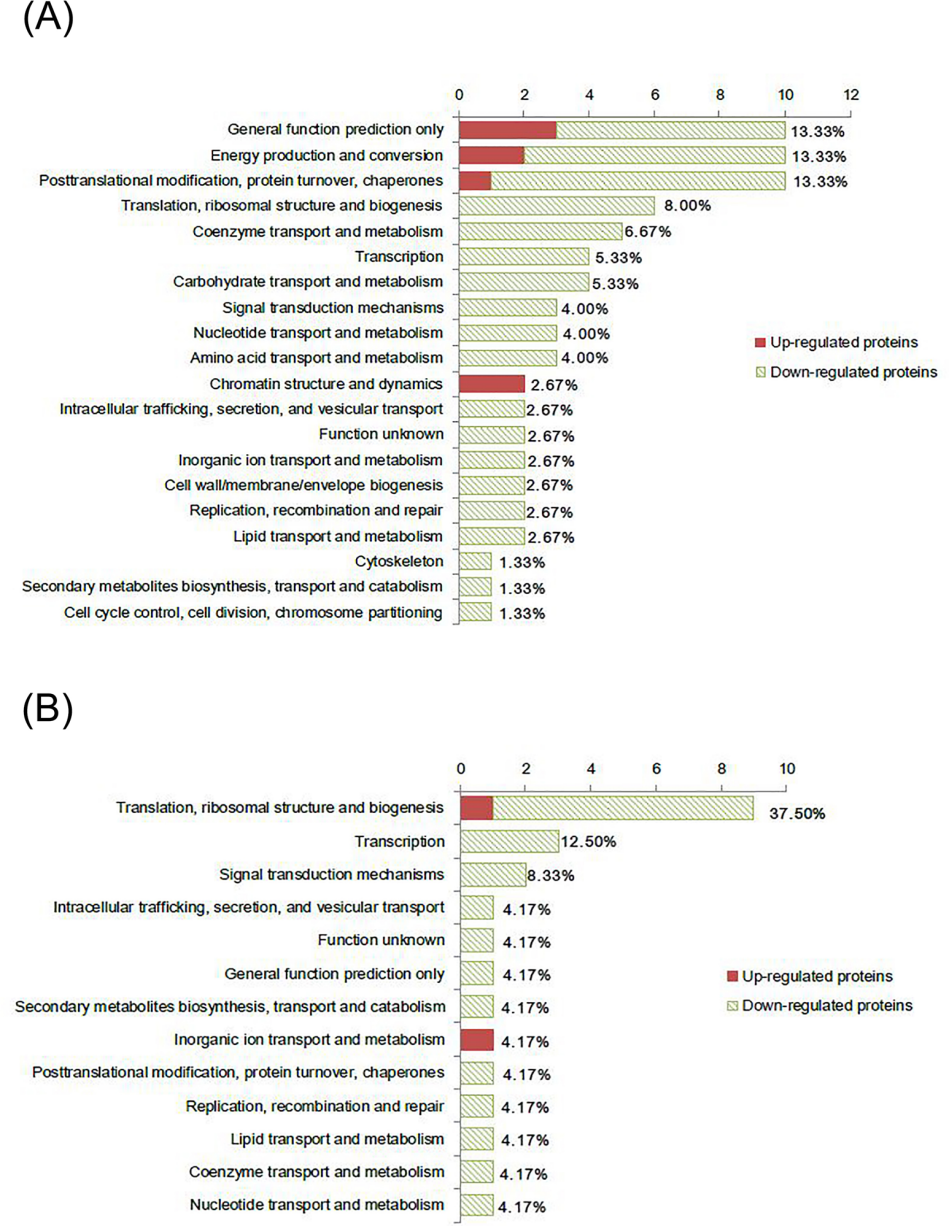
Functional category (COG) coverage of the significantly regulated proteins identified in *T*. *pseudonana* under N-limited (A) and P-limited (B) conditions based on iTRAQ-LC-MS/MS analysis. One protein may be assigned to more than one functional category. Significantly regulated proteins without COG description (58 and 35 proteins in N- and P-limited cells, respectively) are not shown.

COG enrichment analysis was carried out on 64 and 21 proteins for N and P limitation, respectively (7 and 2 proteins had more than one COG annotation, respectively; 58 and 35 SDEPs, respectively, had no COG annotation; Fig 5). Functional classification indicated differences between not only the functional category coverage but also the compositions for individual categories between the two limited conditions (Fig 5). There were 20 and 13 functional categories annotated in N- and P-limited cells, respectively. Based on the number of SDEPs identified in a single functional category, the most frequently detected functional categories in N-limited cells were general function prediction only, energy production and conversion, and post-translational modification, protein turnover, and chaperones (13.33%, 10 proteins in each category; Fig 5A). The top functional category in P-limited cells was translation, ribosomal structure and biogenesis (37.5%, 9 proteins), which contained 1 upregulated and 8 downregulated proteins (Fig 5B).

### Quantitative PCR validation of proteomic analysis

**Fig 6:**
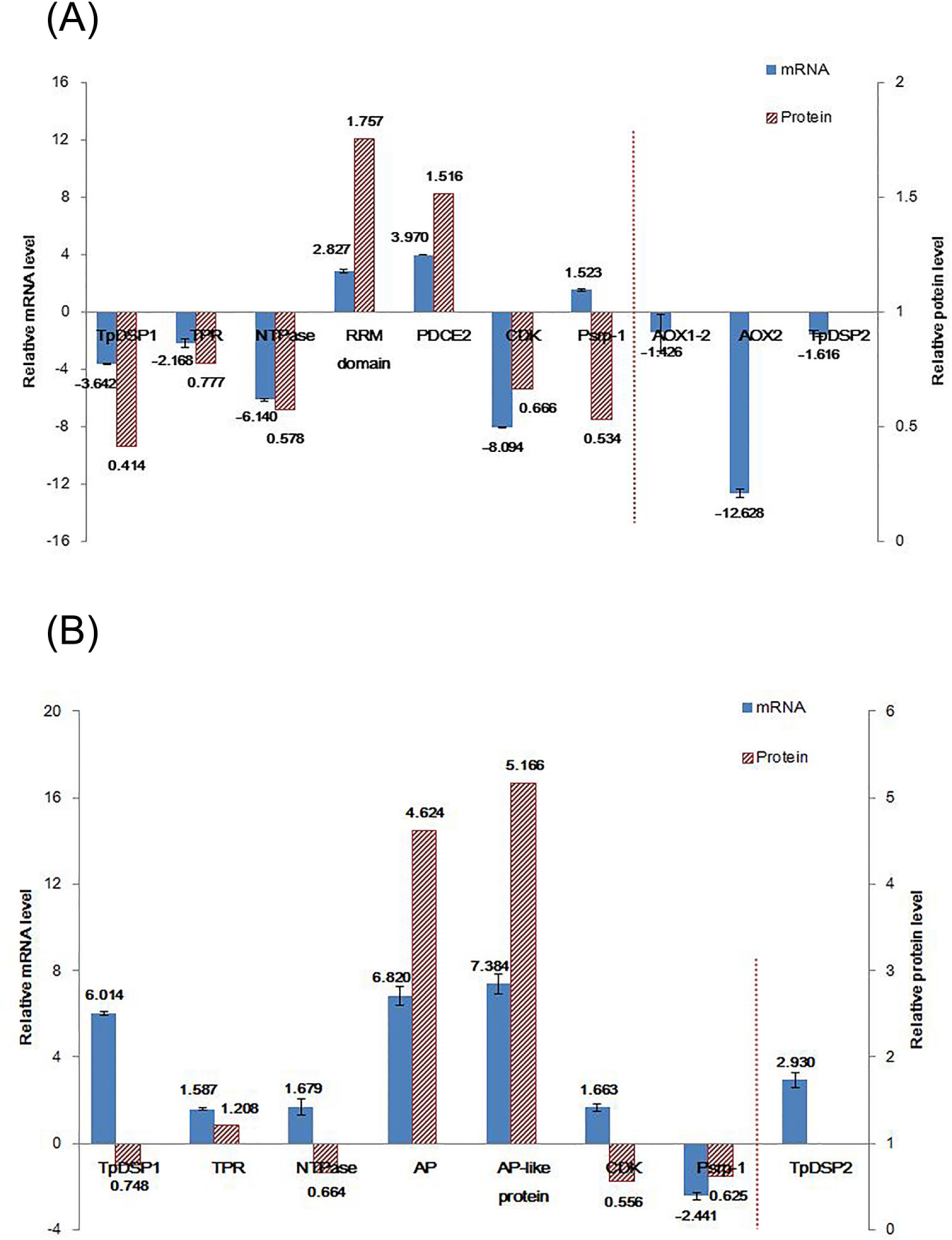
Comparison of mRNA and protein levels of selected genes. Genes include *TpDSP1* (Thaps3_11118), *TPR* (tetratricopeptide repeat protein, Thaps3_22882), *NTPase* (Thaps3_24162), *RRM domain* (RNA-binding proteins, Thaps3_21534), *PDCE2* (pyruvate/2–oxoglutarate dehydrogenase complex, dihydrolipoamide acyltransferase (E2) component, Thaps3_14235), *AP* (alkaline phosphatase protein, Thaps3_1179), *AP-like protein* (Thaps3_1669), *CDK* (cyclin-dependent kinase, Thaps3_20362), *PSrp-1* (ribosome-associated protein Y, Thaps3_255232), *AOX1_2* (Thaps3_38428), *AOX2* (Thaps3_42992), and *TpDSP2* (Thaps3_11117). Translation elongation factor-1α (Thaps3_29435) and ubiquitin-conjugating enzyme (Thaps3_27711) were used as housekeeping markers. Fold changes in the transcript expression for individual genes in N-limited (A) or P-limited (B) cells relative to control are normalized by the geometric mean of the housekeeping markers. The expression of genes unidentified in the present iTRAQ data are indicated in the right of the vertical dotted lines. Left and right vertical axes show relative mRNA and relative protein levels, respectively (mean log_2_ *x*-fold expression ratio ± SD from triplicate measurements).

To validate the quantitative results from the iTRAQ analysis, a subset of 9 genes was selected for quantitative PCR for their involvement in important regulatory processes in response to both types of nutrient stress and their wide range of expression levels. Six (Thaps3_11118, 22882, 24162, 20362, 21534, and 14235) and four (Thaps3_22882, 255232, 1179, and 1669) results were consistent with the iTRAQ data in N- and P-limited cells, respectively; while only one (Thaps_255232) and three (Thaps3_20362, 24162, 11118) were not, respectively (Fig 6). Although it is well known that the correlation between RNA expression and protein abundance is normally weak [37], the present positive correlation between quantitative PCR results and iTRAQ data suggests a good overall quality of this proteomic analysis.

The *T*. *pseudonana* death-specific protein 2 (*TpDSP2*, Thaps3_11117) and two alternative oxidases (*AOX1_2*, Thaps3_38428; *AOX2*, Thaps3_42992) were not identified in the present iTRAQ results. Considering their important roles in oxidative stress and cell fate decision, the mRNA level of these three genes was also analyzed here. All were downregulated in N-limited cells, and *TpDSP2* was upregulated in P-limited cells (Fig 6).

### Comparative expression patterns of proteins related to key metabolic pathways

Because of our particular interest in oxidative stress responses under N and P limitations, we examined the expression patterns of proteins associated with ROS accumulation, cell fate decision, and cellular adaption (S7 Table).

Generally, chloroplast and mitochondria are the main apparatus necessary to produce ROS. Several structural proteins of the photosystem and a series of proteins associated with the mitochondrial electron transport chain and respiratory chain, showed decreased expression, consistent with the elevated ROS accumulation in N-limited cells. Furthermore, key proteins associated with antioxidant defense and damage repair were markedly downregulated. Conversely, no notable oxidative stress was observed in P-limited cells. Significant upregulation of two core photosystem proteins was identified, and only one protein in the mitochondrial respiration chain was downregulated. Proteins related to antioxidant defense and damage repair showed no significant changes in abundance in P-limited cells.

Consistent with the lack of caspase-mediated PCD observed in P-limited cells, there was significant upregulation of a probable serine protease inhibitor. Proteins related to cell death control were downregulated in N-limited cells.

Various expression patterns of the proteins associated with cellular adaption were observed between N- and P-limited cells. For N-limited cells, major differences were found in key proteins associated with intracellular N-containing compound recycling (decreased), the glycolysis pathway (increased) and the tricarboxylic (TCA) cycle (increased). For P-limited cells, however, a marked upregulation of proteins involved in organic phosphate absorption was the main response. Despite these variations in nutrient-specific responses, both limited conditions had a common proteomic response that consisted of a general reduction in a series of proteins involved in chlorophyll biosynthesis and protein synthesis-related proteins.

## Discussion

In the past decade, there have been a considerable number of studies of the fundamental cellular responses of diatoms to N and P stress [4–7]. However, the relationship between N or P limitation and oxidative stress is incompletely understood. In the present study in *T*. *pseudonana*, comparative analysis of the cellular responses to N or P limitation enabled the identification of key cellular processes specifically associated with oxidative stress and cell fate decisions. Physiological and biochemical analyses revealed that N limitation significantly inhibited cell growth rate and photosynthetic efficiency, which further resulted in substantial intracellular ROS accumulation and caspase-mediated PCD activation, consistent with previously reported responses to Fe stress [12]. P stress also substantially inhibited growth rate, but did not result in a significant reduction in photosynthetic efficiency, ROS accumulation, or typical caspase-mediated PCD activation.

The similarities in physiological and proteomic responses under the two conditions suggest that some pathway responses are shared by diatoms under different nutrient stresses. However, the differences in physiological and biochemical responses, which were supported by whole cell proteomic data, indicate that fundamentally different metabolic mechanisms are employed by *T*. *pseudonana*. The differences include those in metabolic mechanisms involved in ROS accumulation; cellular responses to oxidative stress and cell fate decisions; and cell acclimation strategies in response to nutrient stresses.

### Metabolic mechanisms involved in ROS accumulation

#### Oxidant damage and ROS production from chloroplasts

Under N-limited stress, the increased abundance of two fucoxanthin chlorophyll a/c proteins (pigment-binding proteins, or FCPs), which belong to the LHCA clade (Thaps3_18228, NCBI description, 1.78-fold) and LHCX in diatoms (green algal LI818-like clade, Thaps3_12096, NCBI description, 1.32-fold), may be associated with the overproduction of ROS in chloroplasts (their roles in excess light energy dissipation have been documented in previous studies [9,38,39]). In addition, upregulation of FCP gene and protein expression was found in response to oxidative stress caused by Fe limitation in the diatoms *T*. *oceanica* [9] and *P*. *tricornutum* [11], as well as in *T*. *pseudonana* [12].

**Fig 7:**
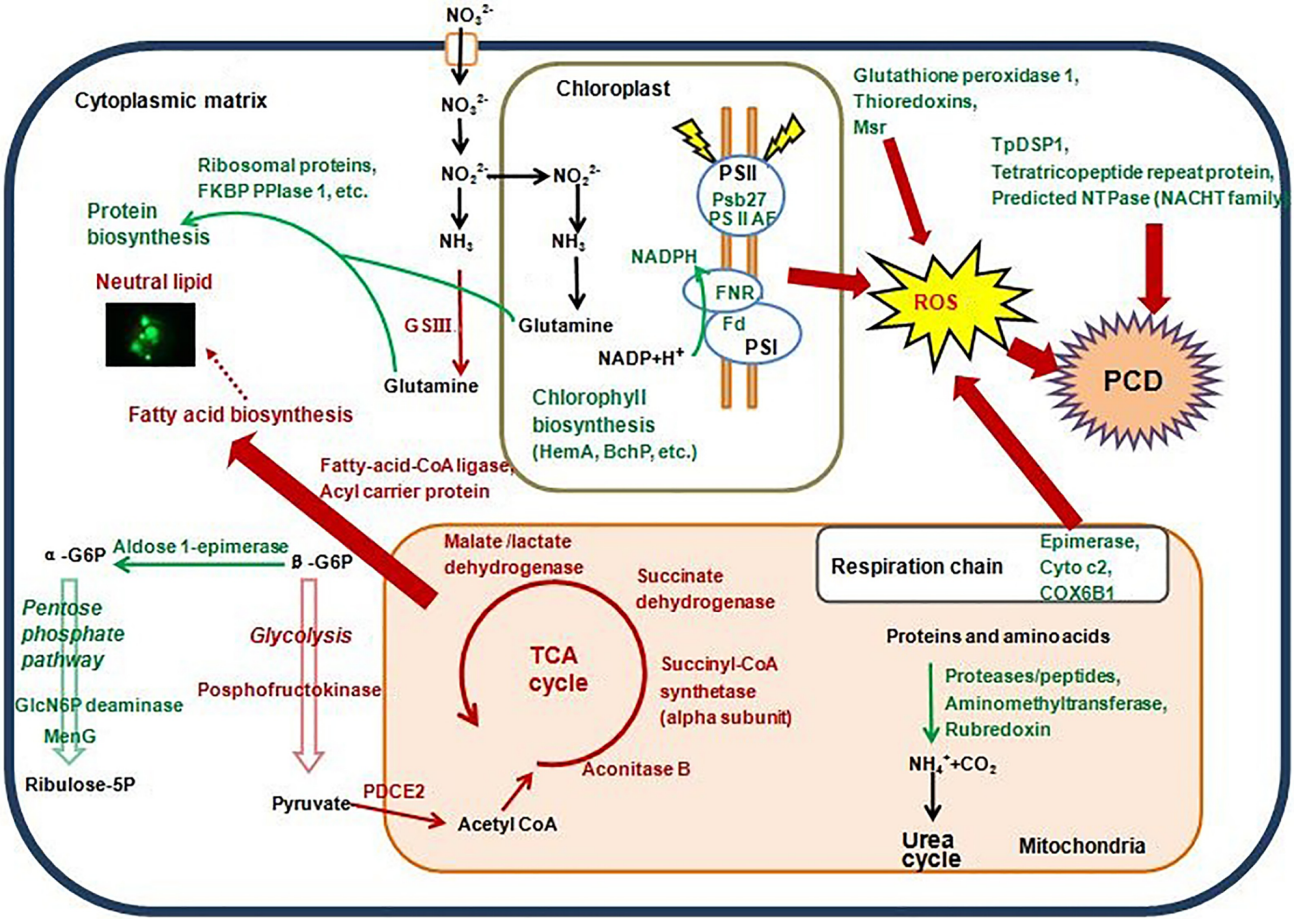
Hypothetical cellular pathways and processes in the diatom *T*. *pseudonana* under N-limited growth conditions. Red words and arrows represent proteins with increased abundance and enhanced pathways, respectively. Green words and arrows represent proteins with decreased abundance and inhibited pathways, respectively. Fluorescence micrograph shows the neutral lipid accumulation in N-limited cells. HemA: porphobilinogen deaminase; BchP: dehydrogenases (flavoproteins); Psb27: photosystem II Psb27 protein; PSII AF: uncharacterized protein related to plant photosystem II stability/assembly factor; Fd: ferredoxin; FNR: ferredoxin-NADP reductase; Cyto c2: cytochrome c2; COX6B1: cytochrome c oxidase subunit 6B1; ROS: reactive oxygen species; Msr: peptide methionine sulfoxide reductase; PCD: programmed cell death; TpDSP: *T*. *pseudonana* death-specific protein; FKBP PPIase 1: FKBP-type peptidyl-prolyl cis–trans isomerase 1; GlcN6P deaminase: glucosamine-6-phosphate isomerase; MenG: demethylmenaquinone methyltransferase; PDCE2: pyruvate/2-oxoglutarate dehydrogenase complex, dihydrolipoamide acyltransferase (E2) component; TCA: tricarboxylic acid cycle; GSIII: uncharacterized protein related to glutamine synthetase (GSIII).

The decreased abundance of the structural proteins under N-limited stress could cause impaired photosynthetic efficiency of PS I and II and ultimately lead to overwhelming oxidative stress and overproduction of ROS (Fig 7). Specifically, PS II Psb27 protein (Psb27, KEGG description, Thaps3_3258, 0.43-fold) is a lumen-localized extrinsic lipoprotein of PS II [40] that facilitates the correct binding and formation of water-oxidizing complexes in chloroplasts [41]. Previous results have demonstrated the essential roles of Psb27 in damage repair [42] and cell survival under stress conditions such as nutrient deprivation [43]. Furthermore, ferredoxin (Thaps3_bd_1258, 0.33-fold) normally functions as an electron carrier/donor in the photosynthetic electron transport chain in PS I, and ferredoxin–NADP reductase (Thaps3_25892, 0.51-fold) is the last enzyme in the electron transfer chain that takes place during photosynthesis; thus, their decreased expression may block electron transfer in photosynthesis.

In contrast, under P-limited stress, the elevated abundance of two core photosystem proteins (photosystem I P700 chlorophyll a apoprotein A2, Thaps3_bd_1249, 1.56-fold; and photosystem Q (B) protein, Thaps3_bd_1045, 1.64-fold) could contribute to the maintenance of photosynthetic efficiency. FCPs showed no significant change in protein abundance. This, with the undamaged photosynthetic efficiency (*F*_*v*_*/F*_*m*_ of ~0.57, >0.5) in P-limited cells, suggests that there is a compensatory mechanism that prevents the photosystem and electron transport chain from damage when the diatom is exposed to P limitation.

#### Oxidative damage and ROS production from mitochondria

A predicted nucleoside diphosphate sugar epimerase (Thaps3_4718), similar to an NAD-dependent epimerase/dehydratase found in the cyanobacterium *Anabaena variabilis*, was also decreased in abundance (0.55-fold). Epimerases are used by the NADH molecule that is produced in the TCA cycle to donate two electrons to the transport chain when NADH binds to the mitochondrial respiratory chain complex I [44]. Thus, inhibition of this protein could lead to a reduced electron supply to the mitochondrial respiratory chain in N-limited cells.

Cytochrome c oxidase of complex IV is a terminal electron acceptor in the mitochondrial respiratory chain, which is involved in transferring electrons from cytochrome c to molecular oxygen and reducing oxygen to water [45]. Under N limitation, a decrease in protein abundance of cytochrome c2 (Thaps3_19927, 0.64-fold) and a possible cytochrome c oxidase subunit 6B1 (COX6B1, Uniprot_Swissprot description, Thaps3_1996, 0.58-fold) could result in a disruption of oxygen reduction and a blockage in mitochondrial electron transport in complex IV, and could therefore also induce an increase in ROS generation (Fig 7). Although overproduction of ROS might also be related to blocking the respiratory system electron transport chain in Fe-limited *T*. *pseudonana* cells [12], the blocked electron transport chain in mitochondria was proposed to result primarily from the inhibited complex III under Fe limitation [12], different to that observed under N limitation.

Unlike N-limited and Fe-limited [12] *T*. *pseudonana* cells, P-limited cells showed no significant changes compared with control conditions in the abundance of cytochrome c or other proteins related to the mitochondrial respiratory chain, except in COX6B1 (Thaps3_1996, 0.67-fold). This is in accordance with the lack of significant ROS production under P limitation.

### Cellular responses to oxidative stress and cell fate decisions

#### Impairment of antioxidant defense systems

Generally, cells have developed antioxidant defenses and damage removal or repair systems to combat oxidative stress [46]. Primary antioxidant enzymes including superoxide dismutase (SOD), catalase and peroxidase were reported to protect against oxidant damage in direct repair systems [46]. In the present study, however, SOD (Thaps3_18050, 1.15-fold) was shown to have just a slight increase in abundance under N-limited conditions, and the abundance of a glutathione peroxidase (Uniprot_Swissprot description, Thaps3_3233, 0.52-fold) was decreased significantly, suggesting that antioxidant enzymes may not be effective ROS scavengers in N-limited cells.

Methionine (Met) residues are endogenous antioxidants in proteins and constitute an important antioxidant defense mechanism [47]. As one of the most sensitive amino acids to ROS, Met is susceptible to oxidation to methionine sulfoxide (MetO) under elevated ROS levels [48], which causes protein dysfunction or aggregation [49]. MetO can be reduced back to Met, and ROS can be eliminated simultaneously, with the help of two distinct stereospecific methionine sulfoxide reductases (Msr), namely MsrA and MsrB [50]. In the present study, the abundance of the Msr peptide (Thaps3_17816) was decreased (0.55-fold) in N-limited cells, revealing that the antioxidant system via Met residues may be impaired. The abundance of Thioredoxin (Thaps3_21965, 0.50-fold) was also decreased. Thioredoxins are required for MsrB-reducing activity [50–52] and are involved in protecting yeast [53] and plants [54,55] against oxidative stress. Hence, it seems that antioxidant systems are ineffective against ROS stress under N-limited conditions. This is different from the response of *T*. *pseudonana* cells to Fe-limited conditions [12], in which antioxidant capacity is maintained by the recruitment of enzymes that do not require Fe.

In addition to the classic respiration pathway, plant mitochondria have an alternative respiratory pathway containing a unique protein, alternative oxidase (AOX), located in the mitochondrial inner membrane [56,57]. Previous studies have demonstrated that an increase in the amount of AOX protein and/or mRNA were involved in mitigating mitochondrial ROS production and enhancing tolerance to abiotic stress in higher plant and Fe-limited *P*. *tricornutum* cells [11,56,57]. However, AOX was not detected in N- or P-limited (present study) or Fe-limited [12] *T*. *pseudonana* cells. The likely reason for this is because the expression level of AOX protein was too low to be detected, or possibly silenced altogether. However, it is reasonable to hypothesize that AOX protein might not contribute to the elimination of ROS accumulation in mitochondria in N-limited *T*. *pseudonana* because of the marked reduction in mRNA expression of *AOX*2 (a *T*. *pseudonana* AOX encoding gene) detected on day 4 based on our quantitative PCR validation (Fig 6).

In P-limited *T*. *pseudonana* cells, however, there was no change in levels of proteins associated with antioxidant defenses or damage removal/repair systems, despite a small increase in expression of thiol-disulfide isomerase and thioredoxins (Thaps3_23961, 1.21-fold) as a result of low cellular ROS accumulation.

#### PCD activation and related proteins

According to previous observations in plants, overproduction of cellular ROS caused by environmental stresses can generate PCD activation signals [58]. In the present study, PCD activation was found in N-limited but not P-limited cells, and *in vivo* staining revealed a high percentage of cells positive for PCD markers corresponding to a high percentage of cells positive for markers of ROS accumulation (Fig 3).

*In silico* research has demonstrated that several cell death-related domains and proteins previously thought to be restricted to animal or land plants are also present in the genomes of some diatom species, including *T*. *pseudonana* [59]. In the present study, two were expressed in N- and P-limited *T*. *pseudonana*. A tetratricopeptide repeat protein (Uniprot_Swissprot description, Thaps3_22882) containing an amino acid sequence for the NB-ARC domain [60,61] was slightly decreased in N-limited (0.78-fold) and mildly increased in P-limited (1.21-fold) cells, corresponding to their mRNA expression patterns (Fig 6). The NB-ARC domain is a signaling motif in plant resistance gene products and regulators of cell death in animals [60,61]. Meanwhile, the protein abundance of an anti-apoptotic predicted NTPase belonging to the NACHT family (Thaps3_24162) [62] was decreased in both N-limited (0.58-fold) and P-limited (0.66-fold) cells. Correspondingly, the mRNA level of the gene encoding NTPase was also decreased in N-limited cells (Fig 6A). The expressions of these cell death-related domains may be involved in the progression of PCD in N-limited cells. The detailed mechanism, regulatory pathways, and precise biochemical roles of these domains in the diatom PCD process require further investigation.

DSP is a recently discovered death-specific protein that was suggested to be involved in self-destructive autolysis in the diatom *S*. *costatum* [63]. Mounting evidence suggests that DSPs have a dual function of acclimation and death facilitation depending on the environmental conditions [13,25]. In the present study, one of two annotated *T*. *pseudonana* DSPs (TpDSP1; NCBI description, Thaps3_11118) showed significant downregulation of protein (0.41-fold) and mRNA levels (-3.64-fold) in N-limited cells (Fig 6A), and a slight decrease in protein abundance (0.75-fold) but significant increase in mRNA levels (6.01-fold) in P-limited cells (Fig 6B). Surprisingly, the similar expression pattern of TpDSP1 in P-limited cells was also observed in Fe-limited *T*. *pseudonana* cells undergoing PCD [12]. According to the previous finding, TpDSP1 is a plastid-targeted protein in *T*. *pseudonana* and its overexpression enhances growth during Fe limitation under low light [13]. The downregulation of this protein in N- and P-limited (this study) and Fe-limited [12] *T*. *pseudonana* cells is consistent with their lower growth rate. Nevertheless, according to another finding that *TpDSP*1 mRNA levels showed a significant increase in response to sub-lethal and lethal ROS levels [13], the expression patterns in our study contradict those observed under Fe-limited conditions [12,13]. Thus, the presence and relevance of this protein in stress acclimation and death facilitation in *T*. *pseudonana* under different nutrient stresses seems more complex than previously thought.

It is noteworthy that a serine protease inhibitor (NCBI description, Thaps3_23814), an important PCD-related protein closely associated with a decrease in caspase-specific activity [64], was identified in this and the Fe limitation study [12]. Its abundance was slightly decreased in N-limited (0.47-fold, *p* > 0.05) and Fe-limited cells [12], but significantly increased in P-limited cells (1.8-fold). The decreased expression in N-limited cells could indicate an increase caspase-specific activity, and that could activate PCD; the increased expression in P-limited cells is consistent with low caspase-specific activity and the lack of caspase-mediated PCD activation identified in P-limited cells.

### Cell acclimation strategies in response to nutrient stresses

To cope with different nutrient-limitation stresses, or different stages of a given stress, diverse and specific acclimation strategies may be employed by diatoms to maintain cell survival [4–7,65–67].

For N-limited cells, a classic nitrate reductase (NR, Thaps3_25299), which is a key protein in the first reduction step of nitrate, was not detected on day 4. This might relate to the nitrate deficiency in the medium, because nitrate is required for the translation of NR mRNA to protein [68]. Meanwhile, the decreased protein abundance of a ferredoxin subunit of nitrite reductase (Thaps3_2673, 0.65-fold) suggests N assimilation is inhibited as a result of the shortage in N. The protein abundance of key enzymes involved in the urea cycle was not significantly changed in N-limited cells. All the observations related to N assimilation metabolism are consistent with the fact that *T*. *pseudonana* was exposed to late-stage N-stress with 24 h of N-starvation (Fig 2B). Corresponding to the severe shortage of N, the synthesis of nitrogenous macromolecules such as chlorophyll a was reduced due to a decrease in abundance of a series of enzymes involved in chlorophyll biosynthesis (S7 Table); the same responses occur in the early stages of N starvation [4]. At the same time, the recycling of intracellular N-containing compounds might be decreased, given that the abundance of several proteins associated with protein degradation and intracellular amino acid metabolism (Thaps3_17419, 12460, 14639, 6836, 15679, 15065, 19155) was significantly decreased by 0.51- to 0.66-fold. In contrast, an increase in abundance of key enzymes in the urea cycle, and enhanced remobilization and redistribution of intracellular N, were employed to respond to early-stage N-starvation in *T*. *pseudonana* [4]. These different responses in the different stages of N stress imply that *T*. *pseudonana* cells generally increase the recycling of intracellular N-containing compounds at the onset of N-starvation. However, such a response might be inhibited as a result of deeper damage during the late stage of N-starvation.

**Fig 8:**
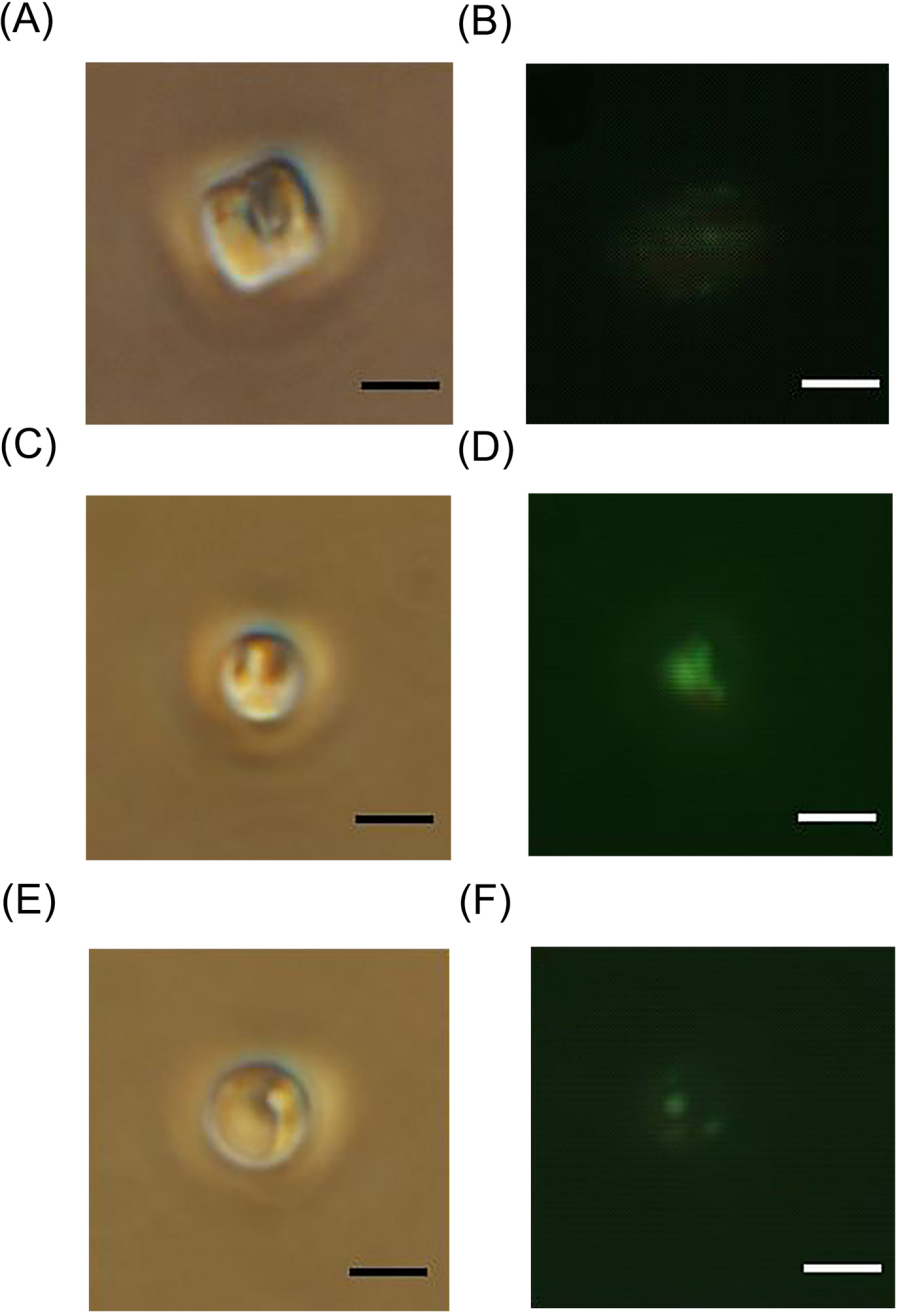
Neutral lipid accumulation in N-limited, P-limited and control *T*. *pseudonana* cells at sampled time points for proteomic analysis (day 4). Fluorescence micrographs (B, D, F) and the corresponding bright-field images (A, C, E) of oil-containing lipid bodies stained with green fluorescent dye (BODIPY 505/515) in cells cultured in control (A, B), N-limited (C, D), and P-limited (E, F) conditions on day 4. Bars: 5 μm.

To address this dilemma, N-limited *T*. *pseudonana* would likely reduce the cell population via PCD to decrease nutrient consumption and stress. Lipid accumulation is another strategy that might be used to survive under stress; this is supported by the observation of *in vivo* neutral lipid accumulation (Fig 8). Proteomic data in the present study should give us an insight into the molecular mechanisms involved in. Under N-limitation, glycolysis pathway might be up-regulated due to the observed increase in abundance of phosphofructokinase (Thaps3_22213, 1.34-fold), a protein associated with the glycolysis pathway (Fig 7); this is also supported by the observed inhibition of the pentose phosphate pathway (pentose and glucuronate interconversion metabolism pathway, Fig 7; [69]), with the significant decrease in protein abundance of an aldose 1-epimerase (Thaps3_1295, 0.59-fold), glucosamine-6-phosphate isomerase/deaminase (Thaps3_10378, 0.59-fold) and demethylmenaquinone methyltransferase (Thaps3_21517, 0.66-fold). Inhibition of this pentose phosphate pathway would facilitate cellular carbon degradation directly through the glycolysis pathway. Furthermore, the significant increase in abundance of two dihydrolipoamide acyltransferases (pyruvate/2-oxoglutarate dehydrogenase complex, dihydrolipoamide acyltransferase [E2] component, PDCE2; Thaps3_2229, 2.22-fold, and Thaps3_14235, 1.52-fold) in the pyruvate dehydrogenase complex (PDHC) may suggest an enhancement of carbon flux from glycolysis into the TCA cycle through acetyl-CoA (Fig 7). Moreover, the abundance of a selection of enzymes involved in the TCA cycle showed an increasing trend; these enzymes include aconitase B (Thaps3_22914, 1.38-fold), the alpha subunit of succinyl-CoA synthetase (Thaps3_28000, 1.42-fold), the flavoprotein subunit of succinate dehydrogenase/fumarate reductase (Thaps3_13214, 1.22-fold) and malate/lactate dehydrogenase (Thaps3_20726, 1.12-fold). Two enzymes involved in fatty acid biosynthesis—a long-chain acyl-CoA synthetase (AMP-forming, Thaps3_1606, 1.49-fold) and an acyl carrier protein (Thaps3_19782, 1.21-fold)—were also increased in abundance. Taken together, the up-regulated glycolysis and PDHC might result in the increased production of carbon precursors and energy for de novo fatty acid synthesis, and enhanced TCA cycle might increase recycling of carbon skeletons derived from degraded proteins and amino acid caused by N-limitation for triacylglycerol synthesis [4,70], which could finally promote lipid accumulation (Figs 7 and 8).

In P-limited cells, the marked increases in protein and mRNA expression of alkaline phosphatase protein (AP protein, NCBI description, Thaps3_1179, 4.62-fold) and AP-like protein (NCBI description, Thaps3_1669, 5.17-fold) suggested that absorption and utilization of organic phosphorus was enhanced. P-limited cells did not suffer from oxidative stress and induce caspase-mediated PCD. We predicted that the utilization of dissolved organic phosphorus to address a deficiency in inorganic phosphorus may represent a critical strategy to maintain metabolic homeostasis in P-limited cells. This hypothesis was consistent with the previous result reported by Dyhrman *et al*. [6]. The previous finding that *T*. *pseudonana* cells can grow substantially under dissolved organic phosphorus alone, and under inorganic phosphorus [71] also supported this hypothesis. The cells can grow very well under a very low concentration of inorganic phosphorus (1/15 phosphate) if the medium contains dissolved organic phosphorus, and grow even better than those under higher concentrations of inorganic phosphorus (1/5 and 1/9 phosphate) [71]. Considering this, we further postulate that a sufficient low concentration of inorganic P-stress may be necessary to trigger an alternative way for the cells to use dissolved organic phosphorus instead of inorganic phosphorus in *T*. *pseudonana*. This hypothesis is partly supported by the previous findings that AP synthesis and AP activity expression were induced only when the concentration of extracellular inorganic phosphorus was below a certain value in some phytoplankton species [72–74], and AP activity expression was co-regulated by extracellular dissolved inorganic phosphorus and intracellular PP [72,75]. However, additional detailed investigations are needed to confirm this hypothesis.

## Conclusions

Our findings highlight the diversity of cellular mechanisms associated with oxidative stress, cell acclimation and cell death control in response to N and P stresses in the diatom *T*. *pseudonana*. The fundamentally different mechanisms of metabolic regulation used under N- and P-limited conditions indicate a high level of flexibility and versatility in the diatom genome and metabolic regulation capability. These findings provide a broad, novel understanding of the potential relationship between the life/death status of the diatom and the markedly different ecological consequences of a highly turbulent marine environment.

## Supporting information

S1 Table Quantitative PCR primers. (XLSX)

S2 Table Full list of identified proteins from *T*. *pseudonana* and their ratios across conditions. (XLSX)

S3 Table COG function classification of the differentially expressed proteins in *T*. *pseudonana* under N-limited conditions. (XLSX)

S4 Table COG function classification of the differentially expressed proteins in *T*. *pseudonana* under P-limited conditions. (XLSX)

S5 Table Proteins showing significantly differential expression in N-limited cells compared with those grown in control conditions. (XLSX)

S6 Table Proteins showing significantly differential expression in P-limited cells compared with those grown in control conditions. (XLSX)

S7 Table Abundance of some important proteins involved in a range of metabolic pathways under N- and P-limited culture conditions. (XLSX)
